# Clinical and experimental approaches for imaging of acute kidney injury

**DOI:** 10.1007/s10157-021-02055-2

**Published:** 2021-04-09

**Authors:** Daisuke Katagiri, Feng Wang, John C. Gore, Raymond C. Harris, Takamune Takahashi

**Affiliations:** 1 Division of Nephrology and Hypertension, Vanderbilt University Medical Center, S-3223 MCN, Nashville, TN 37232 USA; 2 Department of Nephrology, National Center for Global Health and Medicine, 1-21-1 Toyama, Shinjuku-ku, Tokyo, 162-8655 Japan; 3 Vanderbilt University Institute of Imaging Science, Vanderbilt University Medical Center, Nashville, TN USA; 4 Vanderbilt In Vivo Mouse Kidney Imaging Core, Vanderbilt University Medical Center, Nashville, TN USA

**Keywords:** Acute kidney injury, Imaging techniques, Damage biomarkers

## Abstract

Complex molecular cell dynamics in acute kidney injury and its heterogeneous etiologies in patient populations in clinical settings have revealed the potential advantages and disadvantages of emerging novel damage biomarkers. Imaging techniques have been developed over the past decade to further our understanding about diseased organs, including the kidneys. Understanding the compositional, structural, and functional changes in damaged kidneys via several imaging modalities would enable a more comprehensive analysis of acute kidney injury, including its risks, diagnosis, and prognosis. This review summarizes recent imaging studies for acute kidney injury and discusses their potential utility in clinical settings.

## Introduction

The incidence of acute kidney injury (AKI), a serious and common problem associated with a high mortality rate, is increasing [1–5]. The risk of chronic kidney disease (CKD) and end-stage renal disease (ESRD) increases by 8.8-fold and 3.3-fold, respectively, in surviving and discharged patients with AKI [6]. To enable early AKI recognition and sufficient response, several damage biomarkers in the blood and urine have been identified and evaluated [7].

Imaging techniques targeting renal diseases have recently been developed, with each imaging modality having particular characteristics: some have the advantage of clinical translation, whereas others have the disadvantages of high cost or long scan time [8]. The current review aimed to discuss potentially useful and noninvasive imaging techniques targeting AKI as a comprehensive functional biomarker (Table 1) and to explore the possibility of combining them with novel damage biomarkers. The techniques for imaging AKI are as follows: (1) visualize structural renal abnormalities related to CKD underlying AKI [9]; (2) detect urinary tract obstruction, which accounts for 3–10% of AKI [10, 11]; (3) evaluate renal perfusion, oxidation, apoptosis, and fibrosis in each kidney with three-dimensional spatial information; (4) provide noninvasive assessment of the entire kidneys, including the associated organs such as the renal artery, as opposed to renal biopsy, which is invasive and analyzes only a limited portion of kidneys; and (5) evaluate distant organs such as the lungs because remote organ damage in AKI is well known [12]. Investigating currently available imaging techniques both in clinical and basic settings is worthwhile because most of the imaging techniques can be translated into clinics.

**Table 1:** Currently available imaging techniques for AKI evaluation in clinical or experimental settings

	Evaluation	Modality
Clinical imaging	Renal structure and vasculature	Ultrasonography
		CT
	Inflammation	PET–CT with ^18^F-FDG
Basic imaging	Renal structure and vasculature	Ultrasound
		Micro-CT
		T_1_- and T_2_-weighted MRI
	Renal Pathology	Cationic ferritin-enhanced MRI
	Blood flow, blood volume, and urine flow	Dynamic contrast-enhanced MRI
		Fluorine-19 MRI
		Hemodynamic response imaging
		Pulsed arterial spin labeling
		Renal scintigraphy
	Oxygenation	Blood oxygenation level-dependent MRI
	Metabolism	Magnetic resonance spectroscopic imaging Chemical exchange saturation transfer
		PET-CT with ^18^F-FDG
	Fibrosis	Diffusion-weighted imaging
		Magnetization transfer
		Magnetic resonance elastography
		Spin–lattice relaxation time in the rotating frame
	Sodium imaging	^23^Na MRI
	Molecular imaging	Targeted superparamagnetic iron oxide nanoparticles Targeted microbubble contrast agents Optical molecular probes or reporters

*CT* computed tomography, *FDG* ^18^F-fluorodeoxyglucose, *MRI* magnetic resonance imaging

## Molecular mechanisms of acute kidney disease

Numerous factors affect the outcome of AKI, including insult type, hemodynamic alternations, age, underlying CKD, interventions, and genetic variation [5, 13, 14]. Several therapies for AKI have been developed in an effort to explore targets in pathways; however, no attractive therapies are recommended for use in the AKI setting to date [15]. Biochemically, the degree of hypoxia, oxidative stress, cell cycle arrest, suppression of mitochondrial biogenesis, and epigenetic changes contribute to AKI extension [13]. Renal endothelial injury and dysfunction play a pivotal role in the initiation and extension phases of epithelial injury [16]. In ischemic settings, epithelial cells are unable to maintain intracellular ATP, reducing effective perfusion and eventually leading to necrosis or apoptosis [17]. Infiltrating mononuclear phagocytes play an important role in AKI initiation and progression, as well as tissue repair [18, 19]. Dendritic cells and colony-stimulating factor-1-dependent macrophages produce mediators that induce tissue repair after AKI [20]. Cellular repair processes are initiated and organ integrity is reestablished during the maintenance phase [21].With severe or repeated damage to the renal proximal tubules beyond the adaptive repair potential or in the context of abnormal conditions, chronic inflammation, vascular rarefaction, or glomerular sclerosis will subsequently occur, leading to interstitial myofibroblast development [22]. Fibrosis and scarring with a reduction in renal functional mass confirm post-AKI “progression” [16].

## Clinical imaging approaches for AKI

### Ultrasonography (US)

B-mode US is a noninvasive examination routinely conducted on patients with AKI that provides anatomical information about the kidneys, including size (or echogenicity, to distinguish AKI from CKD), hydronephrosis (to rule out post-renal AKI due to urinary tract obstruction), calcification, and cysts [23–25]. As the incidence of urinary tract obstruction is lower in patients with hospital-acquired AKI than in those with community-acquired AKI [26], it is preferable to consider the risk of hydronephrosis, including recurrent urinary tract infections, prior to the performance of US in hospitalized patients with AKI [27, 28]. US enables Doppler-based assessment of resistive index and pulsatility index, which are associated with renal artery and blood flow disorders and persistent or intrinsic AKI [29, 30]. Both indexes are usually obtained by transabdominal or translumbar US; transesophageal US can also be used to assess the resistive index in patients undergoing cardiac surgery for the evaluation of predictable postoperative AKI [31]. Contrast-enhanced US performed with a microbubble-destruction technique [32] is employed for the assessment of patients with septic shock; considering that reduced blood flow in the renal cortex correlates with AKI severity, contrast-enhanced US may become more accurate in evaluating renal perfusion, as it can detect a 15% reduction in normal renal blood flow [33, 34]. US elastography is a currently developing technique that can evaluate renal stiffness [35]. Derieppe et al. [36] reported that renal cortical stiffness measured by US elastography correlated with proteinuria. Although this “stiffness” includes not only fibrosis but also anisotropy, vascularization, or hydronephrosis [35], US elastography can be translated into the sequential assessment of maladaptive fibrosis after severe AKI.

### Computed tomography (CT)

CT provides accurate images of small organs. Diagnosis of atherosclerotic renal artery stenosis with CT angiography is well known, but it is inapplicable to AKI due to the risk of contrast-induced AKI [37]. Experimentally, micro-CT had been shown in mice to have the potential to noninvasively monitor a transplanted kidney’s configuration and function [38].

### Positron emission tomography (PET)–CT with ^18^F-fluorodeoxyglucose (FDG)

FDG is a tracer for glucose metabolism with a well-established utility in cancer imaging. FDG accumulates in not only tumor cells but also macrophages, lymphocytes, neutrophils, and fibroblasts in inflammatory lesions [39]. The diagnostic performance of PET–CT with FDG for inflammatory conditions such as perirenal abscess, vasculitis, and drug-induced acute interstitial nephritis has been reported [40–44].

## Basic imaging approaches for AKI

### Renal structure and vasculature

#### Ultrasound

An advanced noninvasive ultrasound system can identify microvessels. Chen et al. [45] have recently reported the use of super-resolution ultrasound scan to assess microvascular changes, including size, blood volume (BV), and vessel density, after ischemia–reperfusion injury (IRI)-AKI in live mice.

#### Micro-CT

Micro-CT technology and radiopaque perfusion compounds have enabled the acquisition and three-dimensional quantification of renal vascular structure and volume in preclinical models [46]. A previous study reported a reduction in MECA-32-positive peritubular capillaries from day 1, preceded by fibrosis, in an IRI mouse model [47]. While micro-CT cannot examine the capillary structure, a new contrast agent that can make this possible has recently been developed [48]. The structure of renal capillaries can also be assessed using fluorescence microangiography. Kramann et al. [49] reported the loss of peritubular capillary densities after AKI using fluorescence microangiography with renal artery injection.

#### *T*_1_- and *T*_2_-weighted magnetic resonance imaging (MRI)

The protocols of *T*_1_- and *T*_2_-weighted MRI and magnetization transfer contrast MRI have been optimized to acquire high-resolution, high-contrast imaging data of normal and diseased kidneys. Traditional *T*_1_ or *T*_2_ values on MRI have been investigated in patients with AKI. Previous studies using a unilateral IRI-AKI mouse model reported prolonged *T*_1_ and *T*_2_ values due to cell swelling and interstitial edema in the cortex and outer and/or inner medullary stripes [50, 51].

### Renal pathology

#### Cationic ferritin-enhanced magnetic resonance imaging (CF-MRI)

CF-MRI has been used to assess glomerular number and size. Recent reports have shown that this technique could be used for assessing renal pathology in AKI or AKI to CKD transition, including structural changes in glomeruli and renal lesion [52, 53].

### Blood flow, BV, and urine flow

MRI provides anatomical and functional details. Functional MRI has been developed to noninvasively examine renal function and pathology [54–56].

#### Dynamic contrast-enhanced MRI

Several currently available imaging techniques can evaluate renal blood flow in AKI [57]. Dynamic contrast-enhanced MRI, which uses iron oxide nanoparticles or gadolinium-based contrast agents with low molecular weight, can determine the spatial distribution of renal perfusion and vascular reactivity [55, 58–60]. Of note, single-kidney GFR/split function can be estimated using this method [61]. Nevertheless, gadolinium-based techniques are difficult to apply in the setting of human renal dysfunction because of the risk of nephrogenic systemic fibrosis [62, 63].

Intravascular superparamagnetic iron oxide (SPIO) nanoparticles do not undergo glomerular filtration and have a long plasma half-life. Previous studies reported the use of these nanoparticles for BV assessment in both rat and mouse models, as well as their application to patients with CKD [64–66]. In this context, a recent experimental study has shown that capillary rarefaction is more closely associated with AKI-to-CKD progression than renal fibrosis [67]. This imaging technique may be effectively used for assessing AKI-to-CKD progression and for tracking and monitoring the distribution of specific cells (e.g., mesenchymal stem cells) in the AKI setting [68].

#### Fluorine-19 MRI

In addition to ^1^H MRI, fluorine-19 MRI can be employed to quantify changes in blood partial pressure of oxygen (pO_2_) and BV in kidneys [60, 69]. The application of fluorinated emulsions prepared from perfluorocarbons to examine BV fraction and pO_2_ in the renal microvasculature has been explored using mice with AKI [70].

#### Pulsed arterial spin labeling (ASL)

Pulsed ASL techniques label the arterial blood supplying the tissue of interest by altering its longitudinal magnetization [71, 72], and their utility in assessing tissue perfusion, including in transplanted allografts, without the use of contrast agents has been reported [73]. However, these techniques may have limitations in the AKI setting owing to the long scan time [74]. A previous study showed the application of ASL imaging for the measurement of renal cortex perfusion in an IRI mouse model [75].

#### Renal scintigraphy

Renal scintigraphy with injection of tracers such as ^99m^Tc-mercaptoacetyltriglycine (MAG3), ^99m^Tc-diethylenetriaminepentaacetic acid (DTPA), and ^99m^Tc-dimercaptosuccinic acid (DMSA) can evaluate human renal function [76, 77]. The MAG3 tracer is extracted from the kidney through secretion from the proximal tubules, allowing for the evaluation of renal plasma low, tubular function, and urine excretion [78]. Previous studies using ^99m^Tc-MAG3 dynamics reported long-term alterations in renal function after IRI-AKI in mice [79, 80].

^99m^Tc-DMSA is up-taken by renal tubules, enabling the assessment of renal morphology, structure, and function. Dysfunction of megalin/cubilin results in the cessation of renal uptake of a1-microglobulin-bound 99mTc-DMSA and increased urinary excretion [81]. The use of ^99m^Tc-DMSA and ^99m^Tc-MAG3 for cortex imaging and dynamic renography, respectively, in mice with cisplatin-induced AKI has been reported [82]. ^99m^Tc-DMSA was used to evaluate the effects of remote preconditioning in renal function of IRI kidneys [83].^99m^Tc-DTPA is cleared from the plasma through glomerular filtration and is not absorbed or secreted by the tubules, enabling the measurements of single-kidney GFR.

### Oxygenation

As a result of the lower blood flow in the medulla and the countercurrent arrangement that permits oxygen diffusion from arteries to veins, the ambient pO_2_ in the renal medulla is very low (< 20 mmHg) and is even lower than that in systemic venous blood (~ 40 mmHg). The medullary thick ascending limbs (MTALs) contribute to the osmotic gradient by active sodium reabsorption, which requires much oxygen [84]. Hypoxia reduces the ability of tissues to function (i.e., hypoxic injury) [85]. In particular, segment 3 renal proximal tubule cells are highly sensitive to hypoxia in the AKI setting because of low oxygen pressure [86] associated with high energy consumption by ATP-consuming transporters [87, 88]. Post-AKI interstitial fibrosis with impairment in oxygen diffusion is consistently associated with CKD [89].

#### Blood oxygenation level-dependent (BOLD) MRI

BOLD contrast reflects the presence of deoxyhemoglobin in the bloodstream, which changes the signal of protons from the water molecules surrounding a blood vessel [90]. The ratio of oxyhemoglobin, which has no major magnetic property, to deoxyhemoglobin, which is strongly paramagnetic, is proportional to pO_2_. The BOLD signal is estimated using the transverse relaxation rate (*R*_2_* = 1/*T*_2_*) as an indicator of tissue pO_2_. *T*_2_* relaxation time decreases as the deoxyhemoglobin concentration in the blood increases, followed by a decrease in BOLD signal intensity. The utility of BOLD MRI for measuring renal tissue oxygenation, particularly extracellular oxygen tension such as in the bloodstream, has been reported [57, 91]. BOLD MRI is effective in evaluating changes with pharmacological or physiological manipulations, including furosemide, water load, and vasoactive substances [92, 93]. Additionally, BOLD MRI has been reported in IRI-AKI and CI-AKI models [69, 94, 95]. Hofmann et al. [96] reported that post-AKI changes in *R*_2_* values were induced by several drugs, including indomethacin, radio-contrast media, cyclosporine, and tacrolimus. Furthermore, chronological *T*_2_* and *T*_2_ mapping after IRI-AKI in a rat model has been reported [97]. It has also been applied for allograft status evaluation in kidney recipients [98, 99]. Because BOLD MRI is an overall index of the combined effects of oxygen delivery (renal blood flow, extracellular oxygen tension), oxygen consumption (sodium transport in tubules), hydration status, and arteriovenous diffusion [93, 100], caution should be exercised when interpreting BOLD MRI signals from an ailing kidney in complex situations such as AKI and CKD [101].

The quantitative BOLD approach is based on a mathematical model of BOLD contrast [102] and has been mainly evaluated in neuroimaging, although its ability to assess steady-state local basal oxygen saturation should be useful in monitoring time-dependent renal hypoxia in the AKI setting [103]. Different from conventional BOLD MRI, the quantitative BOLD approach provides more specific parameter SO_2_ to evaluate local blood oxygen saturation. Nevertheless, this approach has not been clinically established because of the long acquisition time required [104]. A functional BOLD MRI technique, called hemodynamic response imaging, in which transient alterations in inspired gases from normoxia to hypercapnia and subsequently to hyperoxia enable the evaluation of renal oxygenation, perfusion, and vascular reactivity, has been reported in AKI and CKD models [105]. In addition to BOLD MRI, Hirakawa et al. [106] have recently reported a new technique for quantifying intracellular oxygen tension in an IRI mouse model by measuring the phosphorescence lifetime of small luminescent molecular probes.

### Tissue injury

#### Diffusion-weighted imaging (DWI)

DWI utilizes the difference in water molecule motion between tissues as the image contrast and can thus be rapidly performed without the administration of exogenous contrast agents. This technique senses changes in water molecule motion at the cellular level and provides qualitative and quantitative information that reflects cell membrane integrity (cell damage), cellularity, fibrosis, and perfusion.

DWI measures the apparent diffusion coefficient (ADC), which provides information on quantitative diffusion properties and the contribution of microcirculation in kidneys [74], and has been reported in animal models of unilateral AKI and contrast agent nephropathy [95, 107–109]. Inoue et al. [110] showed the application of ADC to a group of patients with AKI. Another clinical study indicated its usefulness in evaluating allograft function shortly after kidney transplantation and reported significantly lower ADC in transplanted kidneys undergoing acute rejection or acute tubular necrosis [111].

Diffusion tensor imaging provides diffusion measurements (e.g., fractional anisotropy, axial and radial diffusivity) for the evaluation of diffusion changes in different directions and can be applied to renal allograft assessment, as it provides information on the preferred diffusion direction, directed diffusion degree, and renal microstructure [112]. An intravoxel incoherent motion (IVIM) model based on diffusion-weighted images acquired at different *b*-values has been proposed to separate the effects of blood microcirculation perfusion from those of tissue diffusion [113, 114]. The perfusion fraction in AKI on IVIM imaging is affected by several conditions, including cast accumulation in tubules [115].

### Metabolism

Certain metabolic dysfunctions such as amino acid, purine, taurine, and choline dysregulations have been identified in CKD rat models [116]. Notably, the characterization of certain metabolites in AKI is informative and one of the strengths of the imaging field. For instance, the production of renal fumarate metabolite, which increases in necrotic cells, is detectable with ^13^C magnetic resonance spectroscopic imaging in mice with folic acid-induced AKI [117].

#### Chemical exchange saturation transfer (CEST) imaging

CEST MRI displays the interactions between solute protons including amine, amide, or hydroxyl groups that resonate at specific spectral components and can be used to image important metabolic parameters that change in diabetic kidney disease (e.g., intracellular proteins, pH, levels of metabolites such as glycogen, glycosaminoglycan, and glutamate) [118]. Using a db/db endothelial nitric oxide synthase knockout model, we evaluated the utility of CEST imaging in assessing the progression of diabetic nephropathy and identified CEST effects corresponding to relative glucose/glycogen levels [119]. Longo et al. reported the use of CEST MRI with iopamidol for measuring temporary pH elevation in both kidneys in several AKI mouse models [120, 121]. CEST MRI has also been applied in a lipopolysaccharide-induced septic AKI mouse model [122].

#### ^18^F-FDG PET/CT

Reuter et al. [123] reported a series of ^18^F-FDG accumulations in cases of acute rejection of allogeneically transplanted kidneys ameliorated by immunosuppressive therapy in mice. Furthermore, this PET imaging has been applied to examining delayed graft function and AKI in post-renal transplant patients [124]. While ^18^F-FDG accumulation is not specific to certain cells, ^18^F-FDG labeling of isolated leukocytes can discriminate the rejection of allogeneically transplanted kidneys from IRI, acute cyclosporine toxicity, or responses to syngeneically transplanted kidneys [125].

### Fibrosis

Previous studies investigated CKD progression with persistent interstitial fibrosis, followed by maladaptive processes [126, 127]. Proximal tubule injury beyond the adaptive repair potential will arrest epithelial cells in the G2/M transition of the cell cycle and will enhance the production of profibrotic factors [22, 128]. Repeated or rambling renal damage results in extracellular matrix accumulation and tubular atrophy, eventually leading to hypoxia and interstitial fibrosis in kidneys [67, 129, 130]. Thus, assessing the degree of renal fibrosis in patients with AKI will be crucial to their treatment. DWI or BOLD imaging can be used to assess renal fibrosis [110, 131–133]; however, factors other than fibrosis (e.g., blood flow) can change ADC or *R*_2_*. Hence, a more fibrosis-specific imaging method is required to precisely assess renal fibrosis.

#### Magnetization transfer (MT)

MT can detect large immobile macromolecules distributed within tissues and evaluate pathophysiological events (e.g., fibrosis, apoptosis) accompanied by changes in macromolecular components. We and others have recently shown that renal fibrosis can be assessed using specific parameter pool-size ratio from the quantitative MT approach based on mathematical modeling or MT ratio from simpler two-point metrics [132, 134–136]. Importantly, changes in physiological conditions and hemodynamics can affect measurements on DWI and BOLD imaging, even in the absence of fibrosis. However, MT measures remain the same during functional changes in kidneys [137].

#### Magnetic resonance elastography (MRE)

Similar to US elastography, MRE is an MRI modality that enables visualization of tissue elasticity and is sensitive to fibrotic changes [132, 133]. Nonetheless, its specificity and sensitivity to detect interstitial fibrosis may not be high because other factors such as tubular damage and renal blood flow also affect MRE-derived stiffness [138].

#### Spin–lattice relaxation time in the rotating frame (T1rho)

The use of spin–lattice relaxation time in the rotating frame (T1rho) for renal fibrosis assessment has recently been shown [139, 140]. T1rho imaging is a new MRI technique that can sensitively detect macromolecules, including collagen and proteoglycans [141], and may also be used for renal fibrosis assessment in patients with AKI.

### Sodium imaging

AKI may lead to insufficient oxygen utilization for tubular sodium transport [100]. Sodium (^23^Na) MRI provides a direct measure for determining the tissue sodium concentration (TSC) [142, 143]. In normal kidneys, the sodium signal intensity gradually increases from the cortex to the inner medulla. Atthe et al. [144] reported impairment in sodium reabsorption due to acute tubular necrosis after moderate-to-severe IRI-AKI in rats. TSC was more prominently decreased in the medulla, especially the outer medulla and MTALs, than in the cortex. Marill et al. [142] reported significantly reduced TSC in the inner medulla in a model of contrast medium-induced AKI, in which necrosis was limited to only 4% of MTALs.

### Molecular imaging

Specific molecular events during AKI can be assessed using MRI or ultrasound imaging with targeted contrast agents. Akhtar et al. [145] conjugated a vascular cell adhesion protein-1 (VCAM-1) monoclonal antibody to 1-μm iron oxide microparticles in order to visualize and define the three-dimensional distribution of VCAM-1 expression after IRI-AKI in rats. SPIO nanoparticles are notable in AKI owing to the absence of contrast-induced AKI risk associated with the use of iodinated contrast medium. The use of ultrasound with enzyme-loaded nanospheres to detect oxidative stress after IRI has been reported [146]. Recently, microbubble contrast agents have been improved to ligand-specific monoclonal antibodies such as P-selectin and VCAM-1, which were reported to be evaluated in rats with IRI [147]. Furthermore, optical molecular renal probes sensitive to *N*-acetyl-β-d-glucosaminidase and caspase-3 have been developed for real-time imaging and shown to be useful in detecting the early stage of drug-induced AKI in mice [148]. Photoacoustic Imaging techniques allow us to obtain more deep tissue information compared to traditional optical imaging. The recently reported Fluoro-photoacoustic Reporter is designed to look at the activity of gamma-glutamyl transferase (GGT) that was excreted from damaged tubules in AKI [149].

## Utility of imaging techniques in the diagnosis and treatment of AKI

**Fig. 1:**
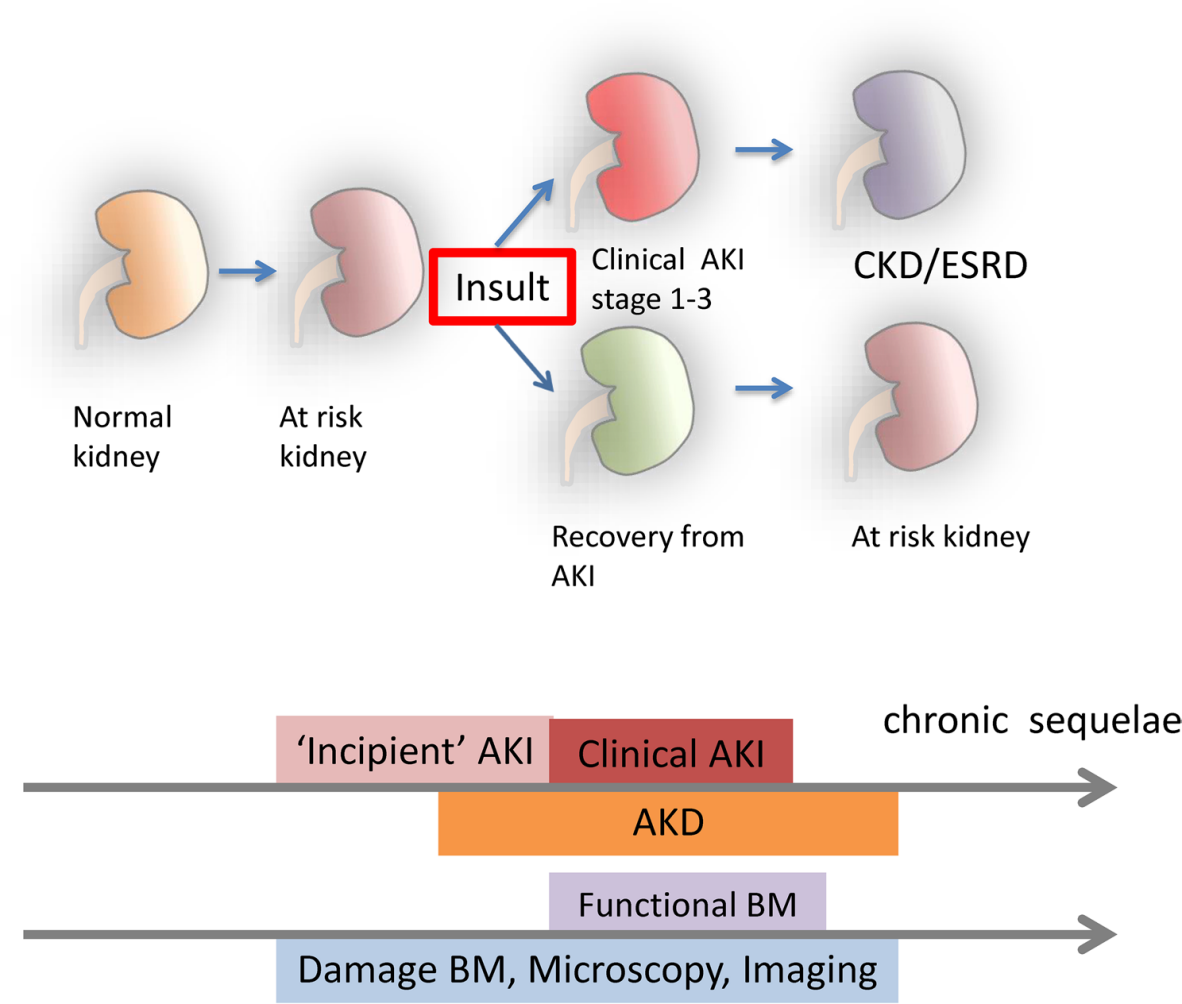
Overall strategy for assessing AKI in different stages. Throughout the course of AKI, patients should be assessed using functional biomarkers (BM) including sCre or BUN, damage BM, urine microscopy, and various imaging techniques

Actions are worthy to be undertaken during the “incipient AKI” phase prior to the occurrence of “clinical AKI,” which is currently based on sCre elevation [150]. The duration of this short-term therapeutic window and the clinical period of AKD, including AKI, are highly important for predicting kidney prognosis [151]. Because the “incipient AKI” phase can lead to diverse outcomes, from full recovery to several clinical AKI stages, it should be carefully investigated using damage biomarkers, urine microscopy findings, and imaging techniques (Fig. 1). Imaging techniques may aid in detecting changes in the microcirculation or tissue oxygenation, and subsequent detection of novel biomarkers may indicate the susceptibility of renal epithelial cells to injury (Table 2). In particular, imaging methods such as CEST imaging or SPIO nanoparticles can help to evaluate sCre-negative but damage biomarker-positive patients with subclinical or incipient AKI [152]. Privratsky et al. [59] reported renal tissue damage detection using dynamic contrast-enhanced MRI after cisplatin treatment from an early stage when sCre and NGAL levels were not elevated. In the context of clinical settings, cutoff values for novel damage biomarkers and imaging information need to be evaluated against patient outcomes (e.g., need for renal replacement therapy) independent of functional biomarkers (e.g., sCre) [13, 153].

**Table 2:** Suggested strategy for assessing responses to AKI: combine novel biomarkers and emerging imaging techniques to detect incipient AKI and evaluate its extension, recovery, or progression to CKD

	Incipient AKI		Clinical AKI	Adaptive repair	Maladaptive repair
	At-risk kidney	Insult, development	AKI extension (or host response)		
Functional biomarkers			Creatinine, urine output		
Damage biomarkers		NGAL, L-FABP, KIM-1	NGAL, L-FABP, TIMP-2, IGFBP-7	MCP-1, UMOD, YKL-40	NGAL, KIM-1
Imaging	US, MRI	BOLD	CEST, ^23^Na MRI, SPIO	CEST, BOLD	qMT, DWI
Others		Sediment	Kinetic eGFR		

*BOLD* blood oxygenation level-dependent, *CEST* chemical exchange saturation transfer, *DWI* diffusion-weighted imaging, *eGFR* estimated glomerular filtration rate, *IGFBP* insulin-like growth factor-binding protein, *KIM-1* kidney injury molecule-1, *L-FABP* L-type fatty acid-binding protein, *MRI* magnetic resonance imaging, *NGAL* neutrophil gelatinase-associated lipocalin, *qMT* quantitative magnetization transfer, *SPIO* superparamagnetic iron oxide, *TIMP-2* tissue inhibitor of metalloproteinase-2, *US* ultrasonography, *MCP-1* Monocyte Chemotactic Protein-1, *UMOD* Uromodulin, *YKL-40* Chitinase-3 like protein 1

Thus far, several large prospective multicenter trials failed to show the sufficient performance of these novel damage biomarkers for clinical use [154–157]. The mechanism of AKI is complex and multifactorial, compelling us to consider the baseline renal function and time duration after kidney insult in heterogeneous patients when applying these damage biomarkers to clinical situations [157]. The performance of each damage biomarker depends on patient populations, timing of measurements, and selected cutoff values. Damage biomarkers themselves reflect the molecular and cellular events in AKI; despite their success in early AKI recognition, problems such as renal prediction and etiologies of AKI are yet to be solved [152, 158]. It is important to choose biomarkers for each different AKI etiology in heterogeneous patient populations. Moreover, few available biomarkers reflect kidney repair/recovery [159].

Imaging techniques provide comprehensive and spatial information about actual pathophysiological events or functional changes in clinical AKI stages. Evaluating the additive value of using imaging techniques as physiological biomarkers along with damage biomarkers of AKI is necessary. Furthermore, we need to consider the effectiveness of imaging techniques for AKI versus the cost and time required, which depends on the modality, before they will be employed in clinical practice.

**Fig. 2:**
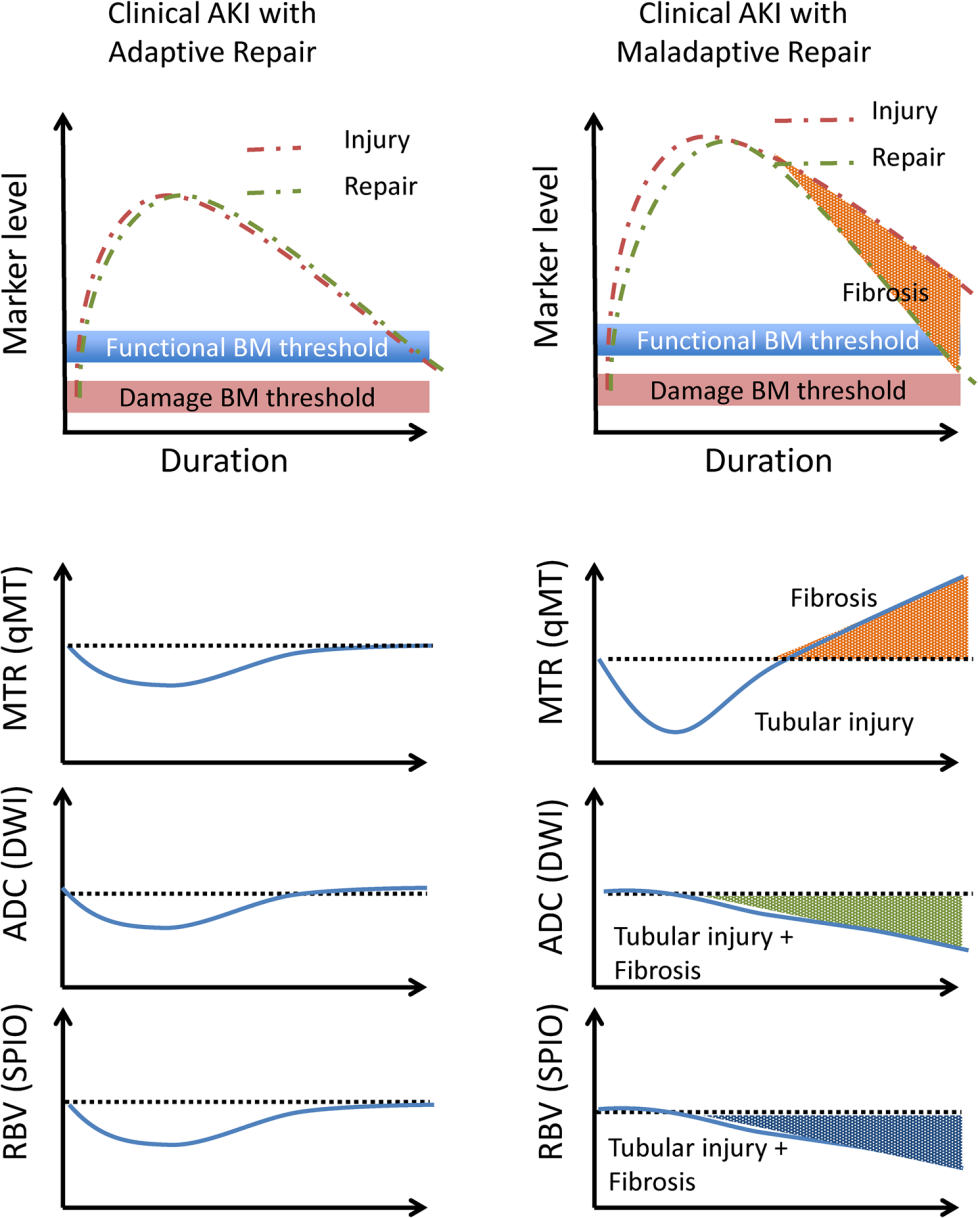
Multi-parametric MRI that may aid in assessing AKI. The top diagams indicate changes in biomarkers (BM) during the course of AKI. The top diagrams were adapted from the *ADQI XIII Work Group. J Am Soc Nephrol. 2015* [13]. The lower charts indicate multi-parametric imaging that can help determine the fibrotic area in maladaptive repair after kidney insult in the absence of an ideal repair biomarker. *qMT* quantitative magnetization transfer, *DWI* diffusion-weighted imaging, *SPIO* superparamagnetic iron oxide

**Fig. 3:**
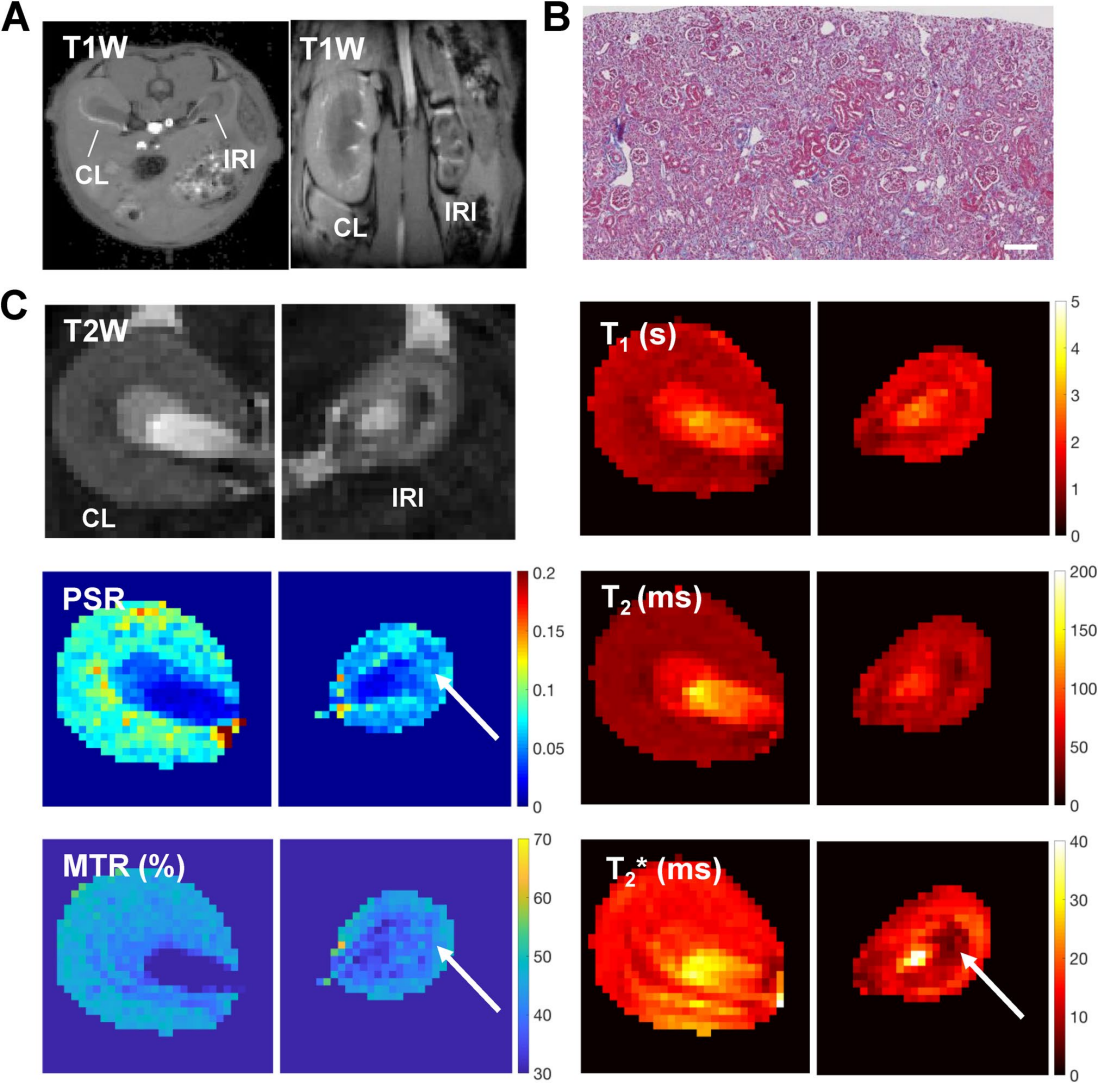
Multi-parametric MRI maps of the mouse kidney after IRI. The left renal pedicle was clipped for 45 min, and MRI was performed at 8 weeks after surgery. (**a**) T_1_-weighted (T1W) anatomical images showing shrinkage of the injured kidney. *IRI* ischemia–reperfusion injury, *CL* contralateral kidney. (**b**) Renal histopathology of the kidney with IRI. Prominent tubular atrophy in the kidney with IRI was observed. Masson’s trichrome staining is shown. Scale bar = 100 μm. (**c**) T_2_-weighted (T2W) anatomical images, T_1_ maps, T_2_ maps, T_2_* maps, pool-size ratio (PSR) maps from quantitative magnetization transfer (qMT) modeling, and magnetization transfer ratio (MTR) maps based on images with and without magnetization transfer saturation (820 degree and RF offset 5000 Hz). PSR and MTR signals and T2* signal intensity is regionally decreased in IRI kidney (arrows); the former indicates renal cell death/atrophy and the latter indicates hypoxia

Patients presenting with positive urinary biomarkers have higher mortality in the long term, even if they do not meet the AKI criteria based on sCre levels or urine output [160]. Given the fact that hyperfiltration of less damaged nephrons could prevent the elevation of sCre, imaging techniques would be quite useful for assessing actual renal conditions of these “biomarker-positive creatinine-negative” patients. Assessing the cause of reduction in several damage biomarkers to evaluate maladaptive repair after AKI would be another condition where imaging techniques are useful [161, 162]. Of note, it may be possible in the future to estimate kidney repair by combined monitoring of appropriate “repair biomarkers”; nevertheless, further translational and clinical investigation should be conducted before we can assess these repair biomarkers [163]. In the absence of available and widely recognized repair biomarkers, the use of current imaging techniques to evaluate the outcomes of AKI, including fibrosis, is reasonable. Several types of information provided by imaging techniques can help us explore the complex pathways of AKI. Among the advantages of imaging are that numerous techniques have already been applied to humans in clinical situations and that multiple parameters can be simultaneously assessed. Therefore, it is important to evaluate AKI using multi-parametric imaging or multiple imaging modalities [134] and to further investigate the value of imaging techniques using animal models (Figs. 2 and 3). Indeed, several recent studies have shown the value of this approach by comparing the findings of multi-parametric MRI with biochemical and/or renal pathology findings in animal models [134] and patients [164–166] with CKD.

## Conclusion

Various new imaging techniques have been developed over the past decade, and their utility in AKI assessment has been shown in preclinical models. Nonetheless, their significance in clinical settings remains largely unknown. Further efforts are required to investigate the utility of imaging parameters or techniques in assessing the time course or pathophysiology of AKI using multi-parametric or multi-modality imaging and to determine their clinical significance by comparing or combining them with currently available biomarkers of AKI. These efforts will further advance our understanding about AKI and improve our treatment protocols.
